# Metabolite Profiling and Pathway Elucidation of 2-Fluorodeschloroketamine and 2-Fluoro-N-ethylketamine in Rats Using HPLC-QTOF Mass Spectrometry

**DOI:** 10.3390/metabo16060394

**Published:** 2026-06-05

**Authors:** Yujie Zhang, Qinghong Wang, Yanjun Wang, Yongfu Wu, Xun Tian, Yong Dai, Yugang Cai

**Affiliations:** 1 Luzhou Public Security Judicial Appraisal Center, Luzhou 646000, China; yujiezhang_995@163.com (Y.Z.); wyf83wfy@163.com (Y.W.); 2 Sichuan Provincial Public Security Department Criminal Investigation Bureau, Chengdu 610000, China; wangqinghong-1@163.com (Q.W.); yanjunwang_cd@163.com (Y.W.); 3 Sichuan Police College, Luzhou 646000, China; xtian@scpolicec.edu.cn

**Keywords:** HPLC-Q-TOF/MS, 2-FDCK, 2-FXE, metabolites, metabolic pathway profiling, metabolic differences

## Abstract

**Highlights:**

**What are the main findings?**

- A HPLC-QTOF/MS system was used to detect potential metabolites of 2-FDCK and 2-FXE in rats, determine metabolic pathways, and analyze metabolic differences.
- After single intraperitoneal injection administration of the drugs, samples were collected at 1 h and 2 h post-dosing. Metabolites were identified through database searches and secondary mass spectrometry fragment ion analysis, allowing inference of metabolic pathways.
- Comparative analysis showed that while metabolic pathways were largely consistent between the two groups, there were significant differences in metabolic reaction types, metabolite formation times, and metabolite response intensities, likely due to chemical structural differences.

**What are the implications of the main findings?**

- The findings offer crucial clues for understanding drug effect disparities between the two compounds.
- They provide a reference for forensic toxicological evaluation of new psychoactive substances in the phencyclidine class.

**Abstract:**

**Objectives**: This study aimed to employ a High Performance Liquid Chromatography coupled with Quadrupole Time-of-Flight Mass Spectrometry(HPLC-QTOF/MS) system to detect potential metabolites of 2-Fluorodeschloroketamine (2-FDCK) and 2-fluoro-N-ethylketamine (2-FXE) in rats, elucidate their metabolic pathways, and analyze the metabolic differences between the two compounds. Such exploration is vital for understanding their distinct drug effects and providing a reference for forensic toxicological assessment of new phencyclidine-class psychoactive substances. **Methods**: Twelve SD rats were randomly split into two groups. After a 12 h fast, each group was administered a single intraperitoneal injection of one drug at 0.045 mg/kg. At 1 h and 2 h post-dosing, the rats were euthanized, and blood, liver tissue, and urine samples were promptly collected. These samples underwent rapid solvent extraction for pretreatment and were then analyzed using HPLC-QTOF. Metabolites were identified through database searches and secondary mass spectrometry fragment ion analysis. **Results**: Comparative analysis of metabolite types, formation times, and chromatographic peak response intensities between the two groups showed that metabolic pathways were mostly consistent. However, significant differences were observed in metabolic reaction types, metabolite formation times, and response intensities, likely stemming from chemical structural disparities. **Conclusions**: The findings offer crucial insights into the drug effect differences between the two compounds and establish a valuable reference for forensic toxicological evaluation of new phencyclidine-class psychoactive substances.

## 1. Introduction

**Figure 1:**
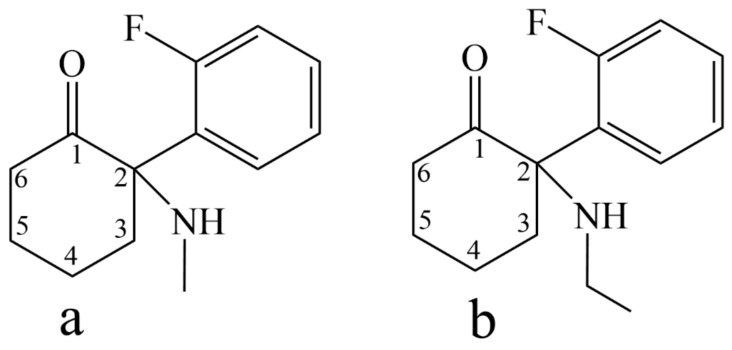
The structure formula of 2-FDCK and 2-FXE. (**a**) 2-FDCK; (**b**) 2-FXE.

2-Fluorodeschloroketamine (2-(2-fluorophenyl)-2-(methylamino)cyclohexan-1-one, 2-FDCK; structural formula in Figure 1a) and 2-fluoro-N-ethylketamine (2-(2-fluorophenyl)-2-(ethylamino)cyclohexan-1-one, 2-FXE; structural formula in Figure 1b) are ketamine derivatives generated through structural modification [1,2,3]. These two drugs fall into the novel new psychoactive substances (NPS) of the phencyclidine (PCP) class and produce central nervous system depressant effects via non-competitive blockade of N-methyl-D-aspartate (NMDA) receptors [4].

As a fluorinated ketamine derivative, 2-FDCK induces distraction, impaired learning ability, and short-term memory deficits at low doses; dissociative hallucinations, perceptual dissociation, and motor control loss at moderate to high doses; and psychosis, disorientation, and amnesia upon high-dose abuse [5,6]. 2-FXE differs from 2-FDCK solely by the substitution of an ethyl group for the methyl group at the N position in its chemical structure [7]. It exhibits highly similar pharmacological effects, though its metabolic process may be modified by this ethyl substitution. Both substances, characterized by low protein binding and high lipophilicity, rapidly cross the blood–brain barrier and are widely distributed throughout the central nervous system.

Both 2-FDCK and 2-FXE exhibit addictive properties. They are frequently encountered in recreational settings, presented as coffee powder mixtures or “euphoric e-cigarettes”. Occasionally, they are co-administered with opioid and barbiturate sedatives, where the synergistic effect of central nervous system depression intensifies respiratory depression and elevates the risk of cardiovascular diseases. Owing to subtle chemical structural alterations, 2-FXE poses greater detection challenges and is employed as a substitute for 2-FDCK to circumvent legal oversight [8].

Currently, research on the two drugs primarily focuses on structural confirmation [1,3], qualitative and quantitative analysis of in vitro samples [9,10], as well as qualitative and quantitative analysis in biological samples [11,12,13] and sewage samples [14,15,16,17], and qualitative and quantitative analysis of metabolites in biological samples [18,19,20]. There have been no reports on the study of the metabolic pathways and metabolic differences between 2-FDCK and 2-FXE. This study provides data support for elucidating toxicological mechanisms, significantly enhances the accuracy and legal validity of drug detection in forensic identification, and serves as a crucial technological approach to addressing the challenges posed by new types of drugs.

In this paper, a rat exposure model was established, and high-performance liquid chromatography-tandem quadrupole-time-of-flight mass spectrometry was employed to detect and analyze the metabolites of 2-FDCK and 2-FXE in rats. The aim was to explore the metabolic patterns of the two drugs in rats, compare the impact of steric hindrance in the two homologs on their metabolic patterns, identify the main metabolic markers, and provide a scientific basis for crimes involving these two substances, which holds significant importance in forensic identification practice.

## 2. Materials and Methods

### 2.1. Instruments and Reagents

Instruments: An Agilent 6546 high-performance liquid chromatography-quadrupole time-of-flight mass spectrometer (Agilent Technologies, Santa Clara, CA, USA), an AL204-IC analytical balance (Mettler Toledo, Greifensee, Switzerland), a BF-2000 nitrogen blow-dry apparatus (Bafang Century, Beijing, China), a Milli-Q Direct 8 ultrapure water system (Millipore, Billerica, MA, USA), and nylon single membranes with a pore size of 0.22 μm (Waters Corporation, Milford, MA, USA) were used.

Reagents: 2-FDCK and 2-FXE ((S)—enantiomers, purity ≥ 99.5%) were sourced from the Shanghai Institute of Forensic Science and Technology (Shanghai, China). Methanol and acetonitrile of HPLC grade were obtained from Fisher Scientific (Waltham, MA, USA), formic acid of HPLC grade from Merck KGaA (Darmstadt, Germany), and anhydrous sodium sulfate of analytical grade from Sichuan Kelong Chemical Co., Ltd. (Chengdu, China).

### 2.2. Instrumental Parameters

#### 2.2.1. Chromatographic Parameters

The chromatographic separation was performed using an Agilent Eclipse Plus C18 RRHD column (3.0 mm × 150 mm, 1.8 μm). The mobile phase consisted of Phase A (0.1% formic acid in water) and Phase B (acetonitrile). The column temperature was maintained at 35 °C, with a flow rate of 0.6 mL/min and an injection volume of 1 μL. The gradient elution conditions are detailed in Table 1.

#### 2.2.2. Mass Spectrometry Conditions

Ion Source: Electrospray Ionization in positive mode (ESI^+^); Sheath Gas Temperature: 350 °C; Flow Rate: 11.0 L/min; Nozzle Voltage: 500 V; Capillary Voltage: 3500 V; Atomizing Gas Pressure: 35 psi; Drying Gas Temperature: 325 °C; Flow Rate: 10.0 L/min; Capillary Outlet Voltage: 110 V; Skimmer Voltage: 65 V; Octupole Rod Voltage: 750 V; Collision Energy (CE): 10–40 V; Data Acquisition Mode: Full Scan; Range: *m*/*z* 50–1000.

### 2.3. Sample Preparation and Extraction

Blood, liver tissue, and urine samples, collected at different time points during animal experiments, underwent pretreatment using liquid–liquid extraction and acetonitrile deproteinization methods, respectively.

For blood and urine samples: Take 1.0 mL of the sample to be tested, add 2.0 mL of acetonitrile, and vortex for 5 min. Centrifuge the mixture at 12,000 r/min to collect the supernatant. Repeat the extraction process twice. Combine the supernatants in a 10 mL centrifuge tube, and evaporate to near-dryness using a nitrogen blower at room temperature. Subsequently, vortex with 300 μL of methanol until complete dissolution, and filter through a 0.22 μm Nylon membrane for analysis.

For liver tissue samples: Weigh 1.0 g of homogenized liver tissue, add anhydrous sodium sulfate, and grind until it resembles dry sand. Add 2.0 mL of acetonitrile and vortex for 5 min. Centrifuge at 12,000 r/min to obtain the supernatant, and repeat the extraction twice. Combine the supernatants in a 10 mL centrifuge tube, and evaporate to near-dryness using a nitrogen blower at room temperature. Vortex with 300 μL of methanol until fully dissolved, filter through a 0.22 μm Nylon membrane, and then set aside for testing.

### 2.4. Establishment of Animal Experimental Model

A total of 12 SPF Sprague–Dawley (SD) rats (male and female equally, 200 ± 10 g) were provided by the Experimental Animal Center of Southwest Medical University. Rats were randomly divided into 2-FDCK and 2-FXE administration groups. After 12 h of fasting, three rats were used at each time point, including one blank control and two treated rats. Treated rats were given a single intraperitoneal injection at a dose of 0.045 mg/kg. Rats were euthanized at 1 h and 2 h after administration, and heart blood, liver tissue, and urine were immediately harvested for analysis. These samples were then processed according to the method outlined in Section 2.3 and tested under the experimental conditions specified in Section 2.2.

### 2.5. Data Analysis

Mass spectrometry data and response intensity data pertaining to potential metabolites of 2-FDCK and 2-FXE were analyzed using Agilent MassHunter Qualitative Analysis software (version 11.0, Agilent Technologies, Santa Clara, CA, USA).

## 3. Results

### 3.1. Analysis of Metabolites of 2-FDCK and 2-FXE in Rats

Drawing on our research group’s previous findings on new psychoactive substances (NPS) of the phencyclidine (PCP) class [3,13,19,20] and relevant literature reports [5,21,22,23], we deduced the potential metabolites of 2-FDCK and 2-FXE, which are presented in Table 2a,b.

After pre-treatment, the animal experimental samples were analyzed according to the instrumental conditions specified in Section 2.2. Agilent MassHunter Qualitative Analysis software was employed for mass spectrometry analysis of the potential metabolites of 2-FDCK and 2-FXE. The precise mass numbers (with an error margin < 5 ppm), isotope distribution patterns, and isotope spacing of the metabolites were extracted from high-resolution mass spectrometry data. Subsequently, elemental composition analysis was conducted through molecular formula derivation. Compounds with a molecular formula matching degree of ≥90% were selected as candidate metabolites using the FBF (Find by Formula) mode. Ultimately, it was confirmed that 2-FDCK and 2-FXE formed nine metabolites each in rats. The precise mass numbers, primary mass spectrometry (MS^1^), and secondary mass spectrometry (MS^2^) fragmentation characteristics of each metabolite are detailed in Table 3 and Table 4. Under the same conditions, analysis of blank and control samples (standard solutions of 2-FDCK and 2-FXE at a concentration of 100 ng/mL) revealed no detection of the metabolites listed in Table 3 and Table 4, indicating that all the listed metabolites were generated through metabolism in rats and were free from endogenous interference.

### 3.2. Mass Spectrometry Analysis

#### 3.2.1. Mass Spectrometry Analysis of 2-FDCK and Its Metabolites

Table 3 summarizes the high-resolution mass spectrometric data for 2-FDCK and its nine predominant metabolites identified in the rat metabolism study. The relative errors between their measured and theoretical [M + H]^+^ *m*/*z* values are all within 1.68 ppm. The corresponding MS/MS spectra and characteristic fragment ion analyses are presented in Figure S1.

##### Mass Spectrometry Analysis of 2-FDCK

Mass spectrometry analysis reveals that 2-FDCK (C_13_H_16_FNO) has a retention time of 5.215 min, with a protonated molecule (*m*/*z*) of 222.1291. The main fragment ions are generated through the following processes: The parent ion loses one molecule of H_2_O, resulting in fragment ions with an *m*/*z* of 204.1179. Alternatively, the aminomethyl group is removed, producing fragment ions with an *m*/*z* of 191.0864. Fragment ions with an *m*/*z* of 204.1179 undergo deaminomethylation, forming fragment ions with an *m*/*z* of 173.0763. The fragment ion with an *m*/*z* of 191.0864 loses one molecule of CO, yielding a fragment ion with an *m*/*z* of 163.0917; or it loses its ethenyl ketone moiety, generating a fragment ion with an *m*/*z* of 147.0602. The fragment ion with an *m*/*z* of 163.0917 loses one molecule of ethylene, creating a fragment ion with an *m*/*z* of 135.0602; or it loses one molecule of butadiene, producing a fragment ion with an *m*/*z* of 109.0449; or it loses the fluorophenyl group, forming a pentadiene cation with an *m*/*z* of 67.0539.

##### Mass Spectrometry Analysis of Primary Metabolites of 2-FDCK

Upon entering the rat body, 2-FDCK is inclined to undergo demethylation metabolism, resulting in the formation of metabolite M09 with the molecular formula C_12_H_14_FNO. The protonated molecule of M09, [(C_12_H_14_FNO) + H]^+^, was detected at a retention time of 4.848 min with an *m*/*z* value of 208.1134. The *m*/*z* values of its principal fragment ions are 191.0869, 173.0764, 163.0919, 147.0608, 135.0607, 109.0450, and 67.0543, which are largely in line with those of the fragment ions of the raw 2-FDCK material.

Two chromatographic peaks with *m*/*z* values of 238.1239 and 238.1242 were observed at retention times of 3.296 min and 4.534 min, respectively. These *m*/*z* values match the protonated molecule [(C_13_H_16_FNO_2_) + H]^+^ of the hydroxylation metabolite C_13_H_16_FNO_2_ of 2-FDCK. These two metabolites, sharing the same molecular formula but differing in retention times, are likely isomers generated from distinct hydroxylation positions, namely M01 and M03. Due to the strong electron-withdrawing effect of fluorine atoms, hydroxylation metabolism on the benzene ring of 2-FDCK is more challenging compared to that on the cyclohexane ring. Moreover, fragment ions with an *m*/*z* of 191.08 were not detected in either metabolite, while fragment ions with an *m*/*z* of 207.08 were identified. Consequently, hydroxylation metabolism of 2-FDCK predominantly occurs on the cyclohexene ring. Theoretically, carbon atoms at positions 3, 4, 5, and 6 on the cyclohexene ring of 2-FDCK are potential sites for hydroxylation reactions. However, the presence of adjacent fluorophenyl and methylamino groups at position 2 creates significant steric hindrance. Simultaneously, the steric hindrance and electron-withdrawing effect of the carbonyl group at position 1 make hydroxylation at positions 3 and 6 on the cyclohexene ring less likely. Therefore, hydroxylation metabolism of 2-FDCK is mainly expected to occur at positions 4 and 5, suggesting that M01 and M03 may be 4-hydroxy-2-FDCK and 5-hydroxy-2-FDCK, respectively. On the hexagonal ring of 2-FDCK molecules, the 5th carbon atom is closer to the carbonyl group. As a result, the hydroxyl group in 5-hydroxy-2-FDCK may be influenced by the electron-withdrawing effect of the carbonyl group, which reduces the electron cloud density of the oxygen atom on the hydroxyl group and facilitates the dissociation of the hydrogen atom, thereby enhancing the molecule’s polarity. Therefore, under the chromatographic conditions described in 2.2.1, the retention time of 5-hydroxy-2-FDCK is shorter than that of 4-hydroxy-2-FDCK, with 5-hydroxy-2-FDCK having a retention time of 3.296 min (designated as M01) and 4-hydroxy-2-FDCK having a retention time of 4.534 min (designated as M03).

##### Mass Spectrometry Analysis of Secondary Metabolites of 2-FDCK

M09 undergoes further deamination to yield metabolite M11, which has the molecular formula C_12_H_13_FO and an *m*/*z* value of 193.1025. Another metabolite, with the formula C_13_H_14_FNO and an *m*/*z* of 220.1132, was identified as M08 based on its MS^2^ fragmentation pattern. M08 is an intramolecular dehydration metabolite of either M01 or M03. Due to steric hindrance, intramolecular dehydration occurs specifically at positions 4 and 5, resulting in M08 as the sole dehydration product of M01 and M03.

The metabolite with the molecular formula C_12_H_14_FNO_2_ and an *m*/*z* of 224.1082 is M17, a hexacyclic hydroxylated metabolite of M09. M09, an N-demethylated metabolite of 2-FDCK, features a substituent group at the 2-position that has changed from methylamino to amino compared to 2-FDCK. The strong electron-donating effect of the amino group significantly alters the electron distribution within the cyclohexanone skeleton, making positions 3 and 6 on the hexacyclic ring the primary sites for hydroxylation. However, due to the presence of substituents at the 2-position, steric hindrance at the 3-position is significantly greater than at the 6-position. Consequently, hydroxylation of M09 likely occurs exclusively at the 6-position of the hexacyclic ring, and no isomer of M17 has been detected, suggesting that M17 may be 6-hydroxynor-2-FDCK.

The metabolite with the molecular formula C_12_H_16_FNO and an *m*/*z* of 210.1290 is identified as M07. Another metabolite, M02, with the formula C_12_H_16_FNO_2_ and an *m*/*z* of 226.1238, has two potential origins. Firstly, it could be a hydroxylated metabolite of M07, with hydroxylation occurring primarily at the 6-position of the hexacyclic ring, similar to the hydroxylation pattern observed in M09, due to the strong electron-donating effect of the amino group and the steric hindrance of the 2-substituent. Secondly, M02 could be a product of carbonyl hydrogenation reduction of M17.

The metabolite with the formula C_12_H_12_FNO and an *m*/*z* of 206.0978 is M05, which is an intramolecular dehydration metabolite derived from the hydroxyl group on the hexanoic ring of M17.

#### 3.2.2. Mass Spectrometry Analysis of 2-FXE and Its Metabolites

Table 4 summarizes the high-resolution mass spectrometric data for 2-FXE and its nine predominant metabolites identified in the rat metabolism study. The relative errors between their measured and theoretical [M + H]^+^ *m*/*z* values are all within 1.46 ppm. The corresponding MS/MS spectra and characteristic fragment ion analyses are presented in Figure S2.

##### Mass Spectrometry Analysis of 2-FXE

Mass spectrometry analysis shows that the retention time of 2-FXE is 5.759 min, and its protonated molecule is *m*/*z* 236.1446. Its main fragment ions include the parent ion losing one molecule of H_2_O to form fragment ions with *m*/*z* of 218.1339, or removing the ethylamine group to form fragment ions with *m*/*z* of 191.0866, and removing the ethylamine group to form fragment ions with *m*/*z* of 173.0762; The fragment ion with an *m*/*z* of 191.0864 loses one molecule of CO to form a fragment ion with an *m*/*z* of 163.0918, or loses its ethylene ketone to form a fragment ion with an *m*/*z* of 147.0605; The fragment ion with *m*/*z* of 163.0917 loses one molecule of ethylene to form a fragment ion with *m*/*z* of 135.0606, or loses one molecule of butadiene to form a fragment ion with *m*/*z* of 109.0449, or loses a fluorophenyl group to form a pentadiene cation with *m*/*z* of 67.0540. The mass spectrometry fragmentation of 2-FXE is basically consistent with that of 2-FDCK, but 2-FXE contains fragment ions of ethylamine group [C_2_H_8_N] + with *m*/*z* of 46.0648.

##### Mass Spectrometry Analysis of Primary Metabolites of 2-FXE

Similar to 2-FDCK, upon entering the rat body, 2-FXE undergoes dealkylation metabolism, yielding metabolite M09_-N_ with the molecular formula C_12_H_14_FNO. The protonated molecule [(C_12_H_14_FNO) + H]^+^ of M09_-N_ was detected at 4.899 min with an *m*/*z* of 208.1134. Both the retention time and protonated molecule matched those of the demethylation metabolite M09 of 2-FXE. The main fragment ions of M09_-N_ exhibited *m*/*z* values of 191.0865, 173.0762, 163.0918, 147.0604, 135.0604, 109.0449, and 67.0541, all consistent with those of 2-FXE’s demethylation metabolite M09.

The cyclohexylated metabolite of 2-FXE, with the molecular formula C_14_H_18_FNO_2_, is analogous to 2-FDCK’s cyclohexylated metabolite. Two chromatographic peaks were identified at retention times of 3.518 min and 4.101 min, respectively. The peak at 3.518 min likely corresponds to 5-hydroxy-2-FXE, while the one at 4.101 min is probably 4-hydroxy-2-FXE The *m*/*z* of 5-hydroxy-2-FXE was measured as 252.1393 (denoted as M01-_N_), and that of 4-hydroxy-2-FXE was 252.1394 (denoted as M03-_N_). The mass spectrometry fragmentation patterns of M01_-N_ and M03-_N_ are largely consistent with those of 2-FDCK’s two cyclohexylated metabolites. Furthermore, fragments of ethylamine ions [C_2_H_8_N]^+^ with an *m*/*z* of 46.0648 are still present in both M01-_N_ and M03-_N_.

##### Mass Spectrometry Analysis of Secondary Metabolites of 2-FXE

The secondary metabolite profile of 2-FXE exhibits a high degree of similarity to that of 2-FDCK. Among these metabolites, M08-_N_ represents the intramolecular dehydration products of M01-_N_ and M03-_N_, characterized by an *m*/*z* value of 234.1289. M09-_N_ undergoes a subsequent deamination reaction, leading to the formation of metabolite M11-_N_, which has a precisely measured *m*/*z* of 193.1024. The cyclohexyl hydroxylation derivative of M09-_N_, identified as M17-_N_, possesses an *m*/*z* of 224.1082 and shares structural and spectroscopic resemblances with the corresponding 2-FDCK metabolite M17. Notably, the hydroxylation process in M17-_N_ specifically targets the 6th position of the cyclohexyl ring, thereby classifying M17-_N_ as 6-hydroxydemethyl-2-FXE. Metabolite M07-_N_ is generated through the hydrogenation reduction of the carbonyl group within M09-_N_, resulting in an *m*/*z* of 210.1290. Another metabolite, M02-_N_, with an *m*/*z* of 226.1238, can be synthesized via two distinct pathways: either through the hydroxylation of M07-_N_ or the hydrogenation reduction of the carbonyl group in M17-_N_. Furthermore, M05-_N_ represents the intramolecular dehydration product of the hydroxyl group present on M17-_N_, exhibiting an *m*/*z* of 206.0979. In terms of mass spectrometry analysis, the fragmentation patterns observed for these 2-FXE metabolites demonstrate a substantial consistency with those of their 2-FDCK counterparts, indicating similar metabolic pathways and structural features.

### 3.3. Metabolic Pathway Analysis of 2-FDCK and 2-FXE in Rats

The metabolites of 2-FDCK and 2-FXE in rats were deduced based on fragment information from secondary mass spectrometry. The primary metabolic pathways of 2-FDCK in rats are illustrated in Figure S3a. Among the nine detected metabolites, three are primary: the cyclohexylated metabolites M01 and M03, and the N-demethylated metabolite M09. The remaining six are secondary metabolites: M08 (dehydration products of M01 and M03), M11 (deamination product of M09), M17 (hydroxylation product of M09), M07 (carbonyl hydrogenation product of M09), M02 (either cyclohexyl hydroxylation product of M07 or carbonyl hydrogenation product of M17), and M05 (intramolecular dehydration product of M17). The metabolic process of 2-FDCK unfolds as follows: initial cyclohexyl hydroxylation followed by dehydration; initial N-demethylation followed by deamination; initial N-demethylation followed by cyclohexyl hydroxylation and then dehydration; initial N-demethylation followed by cyclohexyl hydroxylation and then carbonyl hydrogenation; and initial N-demethylation followed by carbonyl hydrogenation and then cyclohexyl hydroxylation.

The principal metabolic pathways of 2-FXE in rats are depicted in Figure S3b. Among the nine identified metabolites, three are primary: the cyclohexylated metabolites M01-_N_ and M03-_N_, and the N-demethylated metabolite M09-_N_. The six secondary metabolites include: M08-_N_ (dehydration products of M01-_N_ and M03-_N_), M17-_N_ (either N-demethylation products of M01-_N_ and M03-_N_ or hydroxylation product of M09-_N_), M11-_N_ (deamination product of M09-_N_), M07-_N_ (carbonyl oxidation product of M09-_N_), M02-_N_ (further hydroxylation product of M07-_N_), and M05-_N_ (further dehydration product of M02-_N_). The metabolic process of 2-FXE proceeds as follows: initial cyclohexyl hydroxylation followed by dehydration; initial N-demethylation followed by deamination; initial N-demethylation followed by cyclohexyl hydroxylation and then dehydration; initial N-demethylation followed by cyclohexyl hydroxylation and then carbonyl hydrogenation; and initial N-demethylation followed by carbonyl hydrogenation and then cyclohexyl hydroxylation.

In summary, the metabolic pathways of 2-FDCK and 2-FXE predominantly entail hydroxylation, N-dealkylation, carbonyl hydrogenation, and dehydration, processes that augment molecular polarity. Chromatographic analysis reveals that the retention times of the metabolites are shorter than those of the parent compounds, 2-FDCK and 2-FXE, signifying an increase in metabolite polarity during metabolism. These metabolic transformations facilitate drug binding to endogenous substances, such as glucuronic acid, within the body, thereby promoting excretion. However, no metabolites conjugated with endogenous substances like glucuronic acid were detected during the experiments, possibly due to the limited duration of the study. Notably, carbonyl hydrogenation metabolism for both drugs occurred subsequent to N-dealkylation, with no N-dealkylated metabolites of the original drugs being identified. This phenomenon may be attributed to the significant steric hindrance induced by alkyl substitution on the amino group. Additionally, the strong electron-donating effect of the amino group post-N-dealkylation alters the electron cloud density of the cyclohexene ring, a consequence of the combined influence of steric hindrance and electronic effects.

## 4. Discussion

### 4.1. Discussion on the Metabolic Processes of 2-FDCK and 2-FXE

Based on Table 5a, 2-FDCK was detected in the blood at 1 and 2 h post-administration, with a high response intensity, indicating sustained presence of the active ingredient in the bloodstream. The notably high response intensity in urine suggests that some of the active ingredients may be directly excreted via the kidneys. The high response intensity in liver tissue reflects the liver’s robust metabolic activity towards 2-FDCK, designating it as the primary metabolic organ for this compound. Metabolites M01, M03, and M08 were detected only at a single time point, with significantly higher response intensities in urine compared to other metabolites, indicating a faster metabolic rate for intramolecular dehydration following hydroxylation of the precursor, with subsequent renal excretion.

Analyzing the temporal dynamics of 2-FDCK and its metabolites, we observe that from 1 to 2 h, the response intensity of 2-FDCK in urine continues to decline, suggesting a decreasing excretion rate over time. Metabolite M09 exhibits a high response intensity in the blood, which significantly decreases between 1 and 2 h, while maintaining a high intensity in urine, indicating its stability as a metabolite. The response intensity of metabolite M11 in the liver significantly decreases at 2 h, possibly due to further metabolism or excretion. Metabolites M03 and M08 are detected in the liver, while M08 is exclusively found in urine, suggesting they may be tissue-specific metabolites. Metabolite M07, with a high response intensity in urine but undetectable in blood, may be directly metabolized by the liver and excreted without entering the bloodstream. The response intensity of the original amino demethylation metabolite M09 in blood, liver tissue, and urine surpasses that of other metabolites, indicating that N-demethylation is the primary metabolic pathway for 2-FDCK. Following demethylation, 2-FDCK undergoes deamination, cyclohexylation, and carbonyl hydrogenation, resulting in metabolites with low response intensities. Metabolites that undergo further cyclohexylation after carbonyl hydrogenation and dehydration after cyclohexylation are still detectable, albeit with low intensities.

According to Table 5a, after 1 h of administration, the response intensity of the 2-FXE precursor in the blood is relatively high, indicating widespread distribution in the bloodstream within the first hour post-ingestion. By 2 h, the response intensity significantly decreases, suggesting a short half-life and rapid metabolism or excretion of the original drug in the blood. The high response intensity of the active ingredient in the liver at both 1 and 2 h, with a significant increase at 2 h, underscores the liver’s role as the core organ for active ingredient metabolism. The extremely high response intensity in urine, peaking at 1 h and significantly decreasing at 2 h, indicates rapid renal excretion or early prototype excretion of the active ingredient. The original amino demethylation metabolite M09_-N_ exhibits the highest response intensity in urine among all metabolites, indicating that N-demethylation is the primary metabolic pathway for 2-FXE. Following demethylation, 2-FXE undergoes deamination, cyclohexyl hydroxylation, and carbonyl hydrogenation, yielding metabolites with low response intensities. Metabolites that undergo further cyclohexyl hydroxylation after carbonyl hydrogenation and dehydration after cyclohexylation are still detectable, albeit with low intensities. Most metabolites (e.g., M09_-N_, M11_-N_) show significantly higher response intensities in the liver than in the blood, confirming the liver as the core metabolic organ. From a retention time perspective, the original drug has a retention time of 5.7 min, while other metabolites exhibit shortened retention times, possibly involving dealkylation or hydrolysis reactions that increase metabolite polarity. M05_-N_ persists in the liver and urine, suggesting it is an end metabolite suitable for biomarker research.

### 4.2. Comparative Analysis of 2-FDCK and 2-FXE Metabolism

2-FDCK and 2-FXE share structural similarities, differing only in the methyl group on the amino group of 2-FDCK and the ethyl group on the amino group of 2-FXE. The primary metabolic mode for both drugs is N-dealkylation, with dimethyl-2-FDCK (M09, M09_-N_) being the highest responsive metabolite. Thus, dimethyl-2-FDCK serves as the metabolic marker for both drugs. However, differences exist between the two in terms of metabolic rate, metabolite response, and excretion mode. For instance, compared to 2-FDCK, 2-FXE decays faster in the blood and liver, possibly due to accelerated metabolism following ethylation modification. Metabolites of 2-FXE (e.g., M09_-N_) exhibit higher response intensities. Urinary excretion of the 2-FXE precursor is higher at 1 h, but the proportion of metabolite excretion is lower than that of 2-FDCK, reflecting differing renal clearance efficiencies between the two drugs.

### 4.3. Limitations and Interspecies Metabolic Relevance

This study has several key limitations that require clarification. First, the metabolite profiling and metabolic pathway analysis of 2-FDCK and 2-FXE were performed in rats, a classic preclinical animal model. Significant interspecies differences exist in the expression levels, tissue distribution, and catalytic activity of cytochrome P450 (CYP450) enzymes between rats and humans, which may lead to discrepancies in metabolic efficiency, the relative abundance of minor metabolites, and the production of trace metabolites across species. Consequently, the direct extrapolation of the current metabolic findings to humans should be conducted with caution.

Second, this study is an exploratory qualitative metabolic profiling study, aiming at the identification and structural elucidation of metabolites rather than quantitative comparison or statistical analysis. A total of 12 SPF SD rats (male and female equally, 200 ± 10 g) were used and randomly divided into 2-FDCK and 2-FXE groups. At each time point, three rats were employed, including one blank control and two treated rats receiving a single intraperitoneal injection at 0.045 mg/kg. Although biological replicates were included to reduce individual differences, the sample size at each time point remains relatively small and no statistical analysis was performed. The results presented are representative metabolic profiles, which is a common and acceptable strategy for preliminary qualitative metabolite identification in non-targeted metabolomic research.

Despite the above limitations, the core metabolic pathways of the two target compounds identified in rats, including N-dealkylation, hydroxylation, carbonyl reduction, and dehydration, are highly consistent with the well-documented metabolic characteristics of ketamine analogs in humans. Based on the conserved metabolic mechanisms and the present rat data, the primary metabolites expected to be generated in humans are N-dealkylated nor-metabolites, hydroxylated derivatives, and carbonyl reduction products, which can be used as critical biomarkers for the clinical and forensic identification of 2-FDCK and 2-FXE exposure.

This study also provides important practical value for forensic toxicological diagnosis and identification of 2-FDCK and 2-FXE abuse. The N-dealkylated metabolites, hydroxylated metabolites, and carbonyl reduction products were identified as the most abundant and specific metabolites, which show good stability and high signal intensity in biological samples and can be used as priority target biomarkers for forensic screening, qualitative identification, and exposure assessment.

## 5. Conclusions

This study systematically characterized the metabolic profiles of 2-FDCK and 2-FXE in rats using high-performance liquid chromatography coupled with quadrupole time-of-flight mass spectrometry (HPLC-Q-TOF-MS). Key metabolites were successfully identified, and their chemical structures were elucidated. The primary metabolic pathways for both compounds—including hydroxylation, dehydrogenation, and N-dealkylation—were delineated, while the study uniquely demonstrated the differential modulation of metabolic enzyme activity due to steric hindrance effects from adjacent fluorine and ethyl substituents on the benzene ring.

This study also provides important practical value for forensic toxicological diagnosis and identification of 2-FDCK and 2-FXE abuse. Based on the qualitative metabolomic results, N-dealkylated metabolites, hydroxylated metabolites, and carbonyl reduction products were identified as the most abundant and specific metabolites, which show good stability and high signal intensity in biological samples. These metabolites can be used as the priority target biomarkers for forensic screening, qualitative identification, and exposure assessment of 2-FDCK and 2-FXE.

The identified metabolic markers hold potential as biomarkers for biological monitoring, and the comparative analysis of metabolic pathways provides a critical metabolic framework for investigating structure–activity relationships in novel psychoactive substances, such as phenylpyridine derivatives. These findings not only advance the theoretical understanding of 2-FXE disposition in vivo but also offer essential scientific support for toxicity assessment, abuse surveillance, and regulatory oversight of this emerging class of psychoactive compounds.

## Figures and Tables

**Table 1:** Gradient program for HPLC.

Time (min)	A (%)	B (%)	Flow (mL/min)
0.0	70	30	0.6
2.0	50	50	0.6
7.0	30	70	0.6
12	5	95	0.6
17	70	30	0.6

**Table 2:** (**a**) Potential Metabolic Products of 2-FDCK. (**b**) Potential Metabolic Products of 2-FXE.

(a)				
Compound		Formula	Exact Mass	Molecular Weight
2-FDCK	2-FDCK	C_13_H_16_FNO	221.1216	221.28
M01	Hydroxy-2-FDCK (Isomer 1)	C_13_H_16_FNO_2_	237.1165	237.27
M02	Dihydroxy-nor-2-FDCK	C_12_H_16_FNO_2_	225.1165	225.26
M03	Hydroxy-2-FDCK (Isomer 2)	C_13_H_16_FNO_2_	237.1165	237.27
M04	Hydrogenated hydroxyl-2-FDCK	C_13_H_18_FNO_2_	239.1322	239.29
M05	Dehydrated-nor-2-FDCK	C_12_H_12_FNO	205.0903	206.09
M06	Dihydrodeamino-nor-2-FDCK (Isomer 1)	C_12_H_15_FO	194.1107	194.25
M07	Dihydro-nor-2-FDCK (Isomer 1)	C_12_H_16_FNO	209.1216	209.26
M08	Dehydro-2-FDCK	C_13_H_14_FNO	219.1059	219.26
M09	Nor-2-FDCK	C_12_H_14_FNO	207.1059	207.25
M10	Dihydrodeamino-nor-2-FDCK (Isomer 2)	C_12_H_15_FO	194.1107	194.25
M11	Deamino-nor-2-FDCK	C_12_H_13_FO	192.0950	192.13
M12	Dihydro-2-FDCK (Isomer 1)	C_13_H_18_FNO	223.1372	223.29
M13	Dihydro-nor-2-FDCK (Isomer 2)	C_12_H_16_FNO	209.1216	209.26
M14	Glucuronide conjugate of M12			
M15	Dihydro-2-FDCK (Isomer 2)	C_13_H_18_FNO	223.1372	223.29
M16	Dihydrodeamino-nor-2-FDCK (Isomer 3)	C_12_H_15_FO	194.1107	194.25
M17	Norhydroxy-2-FDCK	C_12_H_14_FNO_2_	223.1009	223.25
M18	Glucuronide conjugate of M17			
M19	Glucuronide conjugate of M1			
(**b**)				
**Compound**		**Formula**	**Exact Mass**	**Molecular Weight**
2-FXE	2-FXE	C_14_H_18_FNO	235.1372	235.3
M01-_N_	Hydroxy-2-FXE (Isomer 1)	C_14_H_18_FNO_2_	251.1322	251.3
M02-_N_	Dihydroxy-nor-2-FDCK	C_12_H_16_FNO_2_	225.1165	225.26
M03-_N_	Hydroxy-2-FXE (Isomer 2)	C_14_H_18_FNO_2_	251.1322	251.3
M04-_N_	Hydrogenated hydroxy-2-FXE	C_14_H_20_FNO_2_	253.1478	253.32
M05-_N_	Dehydrated-nor-2-FDCK	C_12_H_12_FNO	205.0903	206.09
M06-_N_	Dihydrodeamino-nor-2-FXE (Isomer 1)	C_12_H_15_FO	194.1107	194.25
M07-_N_	Dihydro-nor-2-FDCK (Isomer 1)	C_12_H_16_FNO	209.1216	209.26
M08-_N_	Dehydro-2-FXE	C_14_H_16_FNO	233.1216	233.29
M09-_N_	Nor-2-FDCK	C_12_H_14_FNO	207.1059	207.25
M10-_N_	Dihydrodeamino-nor-2-FXE (Isomer 2)	C_12_H_15_FO	194.1107	194.25
M11-_N_	Deamino-nor-2-FDCK	C_12_H_13_FO	192.0950	192.13
M12-_N_	Dihydro-2-FXE (Isomer 1)	C_14_H_20_FNO	237.1529	237.32
M13-_N_	Dihydro-nor-2-FDCK (Isomer 2)	C_12_H_16_FNO	209.1216	209.26
M14-_N_	Glucuronide conjugate of M12-_N_			
M15-_N_	Dihydro-2-FXE (Isomer 2)	C_14_H_20_FNO	237.1529	237.32
M16-_N_	Dihydrodeamino-nor-2-FXE (Isomer 3)	C_12_H_15_FO	194.1107	194.25
M17-_N_	Norhydroxy-2-FDCK	C_12_H_14_FNO_2_	223.1009	223.25
M18-_N_	Glucuronide conjugate of M17-_N_			
M19-_N_	Glucuronide conjugate of M1-_N_			

**Table 3:** Mass Spectrometric Profiles of Key 2-FDCK Metabolites in Rats.

Compound	Formula	Retention Time (min)	Mass-to-Charge Ratio of [M + H]^+^ (*m*/*z*)		Relative Error (ppm)	MS/MS Fragments
			Theoretical Value	Measured Value		
2-FDCK	C_13_H_16_FNO	5.215	222.1289	222.1291	0.90	204.1179, 191.0864, 173.0763, 163.0917, 147.0602, 135.0602, 109.0449, 67.0539
M01	C_13_H_16_FNO_2_	3.249	238.1238	238.1235	1.26	220.1127, 207.0816, 189.0710, 179.0870, 161.0761, 147.0604, 123.024, 109.0448, 43.0176
M02	C_12_H_16_FNO_2_	3.102	226.1238	226.1238	0	109.0449, 191.0862, 173.0756, 163.0907, 123.0249
M03	C_13_H_16_FNO_2_	4.534	238.1238	238.1234	1.68	221.0981, 207.0815, 189.0709, 161.0760, 147.0613, 125.0409, 101.0602, 83.0494, 57.0328
M05	C_12_H_12_FNO	4.102	206.0976	206.0978	0.97	189.0715, 171.0605, 161.0761, 146.0524, 133.0449, 109.0443
M07	C_12_H_16_FNO	4.495	210.1289	210.1290	0.48	193.1019, 175.0919, 147.0606, 125.0399, 109.0449, 97.0449
M08	C_13_H_14_FNO	4.755	220.1133	220.1132	0.45	189.0713, 169.0651, 161.0760, 146.0527
M09	C_12_H_14_FNO	4.848	208.1133	208.1134	0.48	191.0869, 173.0764, 163.0919, 147.0608, 135.0607, 109.0450, 67.0543
M11	C_12_H_13_FO	4.500	193.1024	193.1025	0.52	175.0919, 147.0612, 133.0437, 125.0398, 109.0447
M17	C_12_H_14_FNO_2_	4.377	224.1082	224.1082	0	206.0977, 188.0864, 179.0867, 161.0761, 146.0523, 135.0604, 123.0240, 109.0448,

**Table 4:** Mass Spectrometric Profiles of Key 2-FXE Metabolites in Rats.

Compound	Formula	Retention Time (min)	Mass-to-Charge Ratio of [M + H]^+^ (*m*/*z*)		Relative Error (ppm)	MS/MS Fragments
			Theoretical Value	Measured Value		
2-FXE	C_14_H_18_FNO	5.759	236.1446	236.1447	0.42	109.0449, 218.1339, 191.0866, 163.0918, 147.0605
M01-_N_	C_14_H_18_FNO_2_	3.518	252.1394	252.1393	0.40	161.0761, 234.1277, 216.1151, 189.0708, 146.0513
M02-_N_	C_12_H_16_FNO_2_	3.125	226.1238	226.1238	0	109.0448, 191.0877, 173.0763, 163.0916, 123.0240
M03-_N_	C_14_H_18_FNO_2_	4.101	252.1394	252.1394	0	161.0761, 234.1277, 216.1151, 189.0708, 146.0513
M05-_N_	C_12_H_12_FNO	4.113	206.0976	206.0979	1.46	161.0762, 189.0714, 169.0649, 146.0528, 141.0698
M07-_N_	C_12_H_16_FNO	4.522	210.1289	210.1290	0.48	109.0448, 193.1015, 175.0917, 147.0610, 125.0397
M08-_N_	C_14_H_16_FNO	5.319	234.1289	234.1289	0	161.0762, 189.0706, 169.0638, 146.0528, 141.0703
M09-_N_	C_12_H_14_FNO	4.899	208.1133	208.1134	0.48	109.0449, 191.0865, 173.0762, 163.0918, 135.0604
M11-_N_	C_12_H_13_FO	4.517	193.1024	193.1024	0	109.0448, 175.0902, 147.0604, 125.0397, 97.0445
M17-_N_	C_12_H_14_FNO_2_	4.382	224.1082	224.1082	0	109.0450, 206.0968, 179.0864, 161.0761, 135.0604

**Table 5:** (**a**) Main metabolites of 2-FDCK detected in rat’s blood, liver tissue and urine. (**b**) Main metabolites of 2-FXE detected in rat’s blood, liver tissue and urine.

(a)													
Compound	Time of Sacrifice (h)	Retention Time (min)						Intensity					
		Plasma		Liver		Urine		Plasma		Liver		Urine	
		1	2	1	2	1	2	1	2	1	2	1	2
2-FDCK	1	5.216	5.215	5.078	5.202	5.193	5.226	1.1 × 10^6^	2.0 × 10^5^	7.1 × 10^6^	2.5 × 10^5^	9.6 × 10^6^	2.8 × 10^6^
	2	5.108	-	5.083	-	-	-	2.2 × 10^4^	-	3.0 × 10^5^	-	-	-
M01	1	3.262	-	3.249	-	3.296	3.290	1.2 × 10^4^	-	2.1 × 10^4^	-	3.3 × 10^5^	5.8 × 10^4^
	2	-	-	-	-	-	-	-	-	-	-	-	-
M03	1	-	-	4.534	-	-	-	-	-	2.1 × 10^4^	-	-	-
	2	-	-	-	-	-	-	-	-	-	-	-	-
M08	1	-	-	-	-	4.755	-	-	-	-	-	1.6 × 10^5^	-
	2	-	-	-	-	-	-	-	-	-	-	-	-
M09	1	4.907	4.906	4.843	4.866	4.968	4.928	4.0 × 10^6^	2.3 × 10^6^	9.7 × 10^6^	9.6 × 10^6^	9.5 × 10^6^	8.9 × 10^6^
	2	4.872	4.906	4.847	4.847	4.913	4.927	1.0 × 10^6^	8.3 × 10^5^	8.3 × 10^6^	5.8 × 10^6^	2.6 × 10^6^	6.8 × 10^6^
M11	1	4.531	-	4.50	-	4.536	-	1.7 × 10^4^	-	5.0 × 10^5^	-	1.9 × 10^5^	-
	2	-	-	-	4.505	-	4.546	-	-	-	3.4 × 10^4^	-	2.2 × 10^4^
M17	1	-	-	4.377	4.372	4.401	-	-	-	9.8 × 10^4^	8.8 × 10^4^	2.3 × 10^5^	-
	2	-	-	-	4.387	-	-	-	-	-	6.0 × 10^4^	-	-
M07	1	4.531	-	4.495	-	4.536	4.495	1.8 × 10^4^	-	6.1 × 10^5^	-	2.4 × 10^5^	6.3 × 10^4^
	2	-	-	-	-	4.499	4.499	-	-	-	-	2.2 × 10^5^	3.9 × 10^4^
M02	1	-	-	3.108	3.102	-	-	-	-	2.2 × 10^4^	4.4 × 10^4^	-	-
	2	-	-	3.102	3.107	3.129	3.165	-	-	1.0 × 10^5^	4.7 × 10^4^	1.9 × 10^4^	4.7 × 10^4^
M05	1	-	-	4.102	4.102	-	4.126	-	-	1.9 × 10^5^	3.8 × 10^5^	-	3.0 × 10^5^
	2	-	4.114	4.089	4.089	4.122	-	-	2.3 × 10^4^	1.9 × 10^5^	1.3 × 10^5^	5.1 × 10^4^	-
(**b**)													
**Compound**	**Time of Sacrifice (h)**	**Retention Time (min)**						**Intensity**					
		**Plasma**		**Liver**		**Urine**		**Plasma**		**Liver**		**Urine**	
		**1**	**2**	**1**	**2**	**1**	**2**	**1**	**2**	**1**	**2**	**1**	**2**
2-FXE	1	5.748	5.747	5.746	5.723	5.750	5.747	8.7 × 10^5^	4.6 × 10^5^	2.9 × 10^6^	1.1 × 10^6^	8.8 × 10^6^	1.5 × 10^5^
	2	5.740	5.740	5.736	5.737	5.745	5.745	9.9 × 10^4^	1.7 × 10^5^	4.8 × 10^5^	4.0 × 10^5^	1.9 × 10^6^	4.2 × 10^5^
M01-_N_	1	-	3.497	3.518	3.484	3.516	3.503	-	2.6 × 10^4^	2.7 × 10^4^	9.0 × 10^4^	1.6 × 10^5^	2.0 × 10^4^
	2	-	-	3.497	3.493	-	-	-	-	2.9 × 10^4^	3.7 × 10^4^	-	-
M03-_N_	1	4.086	4.092	4.101	4.078	4.116	4.097	1.2 × 10^4^	2.1 × 10^4^	5.2 × 10^4^	2.7 × 10^5^	2.8 × 10^5^	2.0 × 10^4^
	2	-	-	4.081	4.087	-	4.112	-	-	5.3 × 10^4^	9.4 × 10^4^	-	2.9 × 10^4^
M08-_N_	1	5.332	5.332	5.319	5.308	5.346	5.332	2.2 × 10^4^	8.1 × 10^4^	9.7 × 10^4^	1.1 × 10^5^	9.4 × 10^5^	8.9 × 10^4^
	2	5.336	5.341	5.315	5.316	5.347	5.341	1.9 × 10^4^	1.5 × 10^4^	7.0 × 10^4^	2.1 × 10^5^	2.2 × 10^5^	8.0 × 10^5^
M09-_N_	1	4.895	4.894	4.898	4.859	4.936	4.900	4.0 × 10^6^	4.5 × 10^6^	9.6 × 10^6^	9.8 × 10^6^	9.6 × 10^6^	2.0 × 10^6^
	2	4.898	4.898	4.872	4.879	4.932	4.909	2.8 × 10^6^	1.2 × 10^6^	9.8 × 10^6^	9.6 × 10^6^	9.7 × 10^6^	9.3 × 10^6^
M11-_N_	1	4.524	4.513	4.517	4.488	4.532	-	1.4 × 10^4^	1.4 × 10^4^	1.4 × 10^5^	1.9 × 10^5^	1.3 × 10^5^	-
	2	4.522	-	4.496	4.503	4.539	4.528	1.3 × 10^4^	-	1.4 × 10^5^	1.9 × 10^5^	5.9 × 10^4^	3.9 × 10^4^
M17-_N_	1	-	-	4.382	4.353	4.403	-	-	-	2.3 × 10^5^	2.5 × 10^5^	3.1 × 10^5^	-
	2	-	-	4.367	-	4.410	-	-	-	1.4 × 10^5^	-	2.5 × 10^5^	-
M07-_N_	1	-	-	4.522	4.494	4.532	-	-	-	1.7 × 10^5^	2.3 × 10^5^	1.6 × 10^5^	-
	2	4.511	-	4.496	4.497	-	-	1.2 × 10^4^	-	1.6 × 10^5^	2.4 × 10^5^	-	-
M02-_N_	1	-	-	3.125	-	3.129	-	-	-	8.0 × 10^4^	-	1.8 × 10^5^	-
	2	3.108	-	3.093	3.094	3.136	3.113	1.2 × 10^4^	-	1.2 × 10^5^	1.4 × 10^5^	1.5 × 10^5^	1.2 × 10^5^
M05-_N_	1	-	4.109	4.113	4.084	4.128	-	-	9.3 × 10^4^	2.4 × 10^5^	5.9 × 10^5^	1.1 × 10^6^	-
	2	4.102	4.101	4.098	4.099	4.135	4.123	4.9 × 10^4^	3.8 × 10^4^	2.5 × 10^5^	3.6 × 10^5^	4.5 × 10^5^	9.6 × 10^5^

Note: “-” indicates not detected.

## Data Availability

The original contributions presented in this study are included in the article and Supplementary Material. Further inquiries can be directed to the corresponding authors.

## Supplementary Materials

The following supporting information can be downloaded at: https://www.mdpi.com/article/10.3390/metabo16060394/s1, Figure S1: Secondary mass spectra and fragmental information of main metabolites of 2-FDCK; Figure S2: Secondary mass spectra and fragmental information of main metabolites of 2-FXE; Figure S3: Main metabolic pathways of 2-FDCK and 2-FXE.
