# Supplementary material for: Metabolite Profiling and Pathway Elucidation of 2-Fluorodeschloroketamine and 2-Fluoro-N-ethylketamine in Rats Using HPLC-QTOF Mass Spectrometry
Source: Metabolites. 2026 Jun 5;16(6):394. doi: 10.3390/metabo16060394 (PMC13302822; doi:10.3390/metabo16060394)
Supplement: Supplementary file 1 [file metabolites-16-00394-s001.zip › metabolites-4342674-supplementary.pdf]

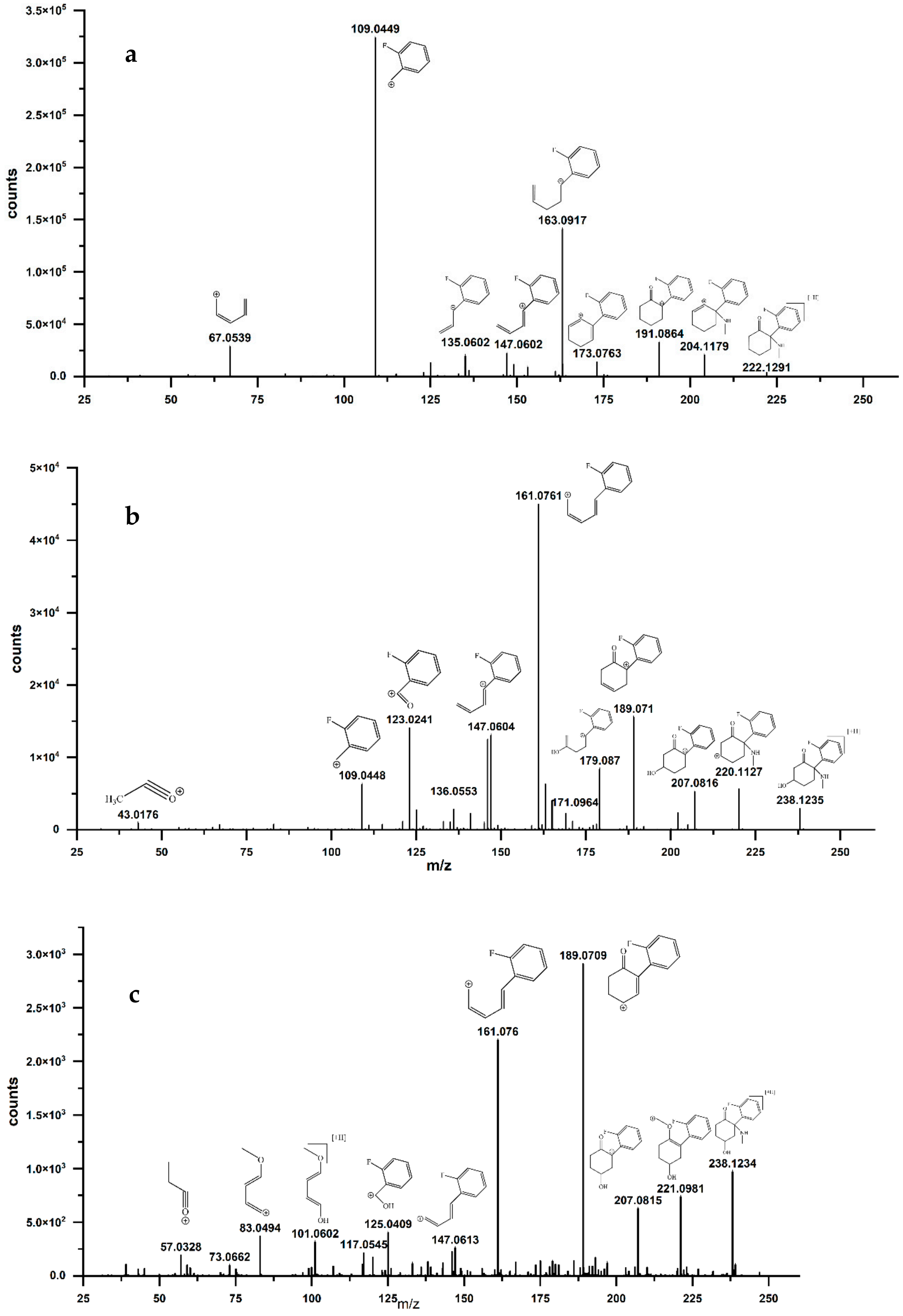

a
counts
3.5×10^5
3.0×10^5
2.5×10^5
2.0×10^5
1.5×10^5
1.0×10^5
5.0×10^4
0.0
109.0449
163.0917
67.0539
135.0602
147.0602
173.0763
191.0864
204.1179
222.1291
25 50 75 100 125 150 175 200 225 250
b
counts
5×10^4
4×10^4
3×10^4
2×10^4
1×10^4
0
161.0761
123.0241
147.0604
189.071
109.0448
179.087
136.0553
171.0964
207.0816
220.1127
238.1235
43.0176
25 50 75 100 125 150 175 200 225 250
m/z
c
counts
3.0×10^3
2.5×10^3
2.0×10^3
1.5×10^3
1.0×10^3
5.0×10^2
0.0
189.0709
161.076
57.0328
73.0662
83.0494
101.0602
117.0545
125.0409
147.0613
207.0815
221.0981
238.1234
25 50 75 100 125 150 175 200 225 250
m/z

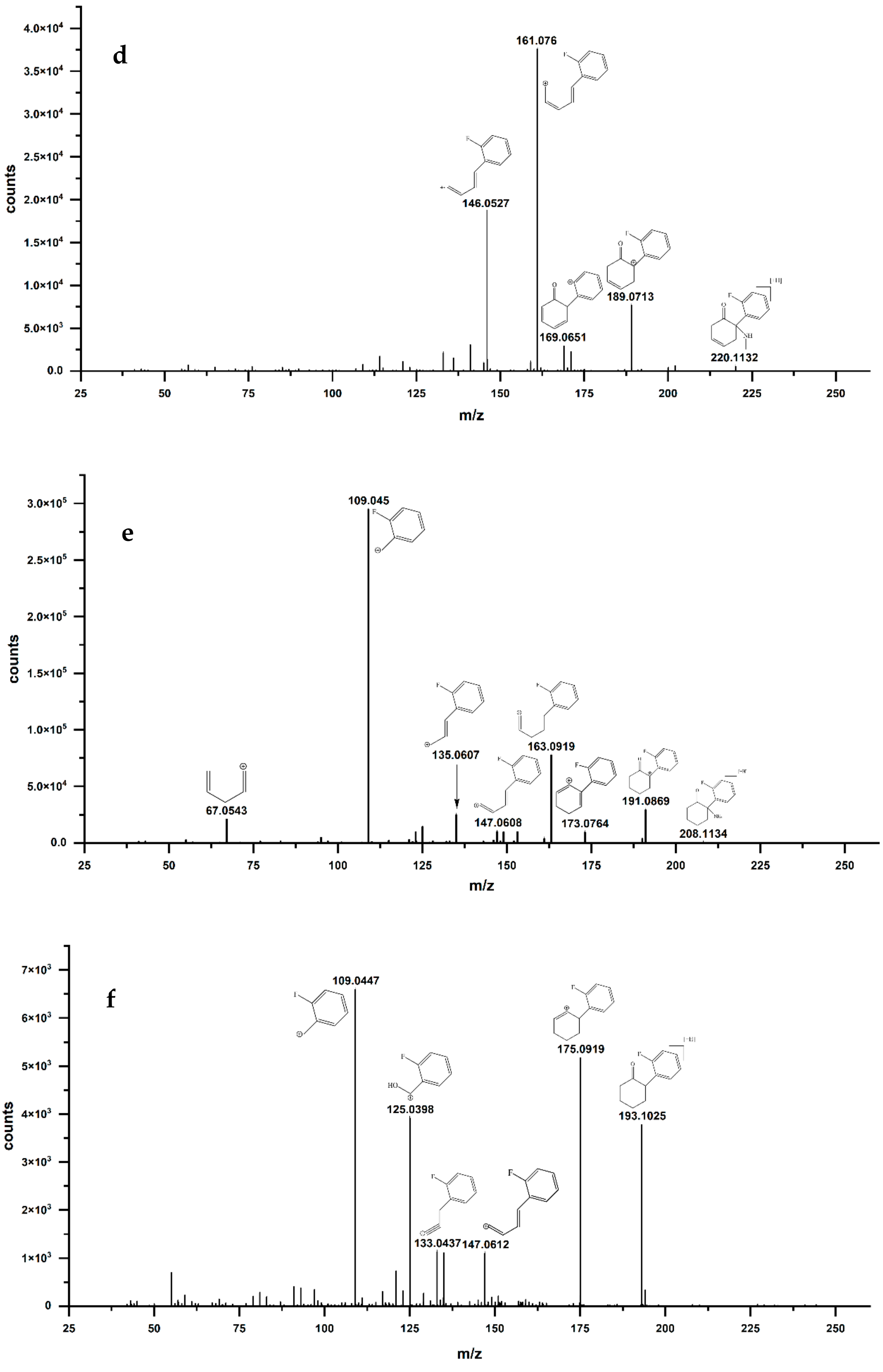
d
161.076
146.0527
169.0651
189.0713
220.1132
counts
m/z
e
109.045
67.0543
135.0607
147.0608
163.0919
173.0764
191.0869
208.1134
counts
m/z
f
109.0447
125.0398
133.0437
147.0612
175.0919
193.1025
counts
m/z

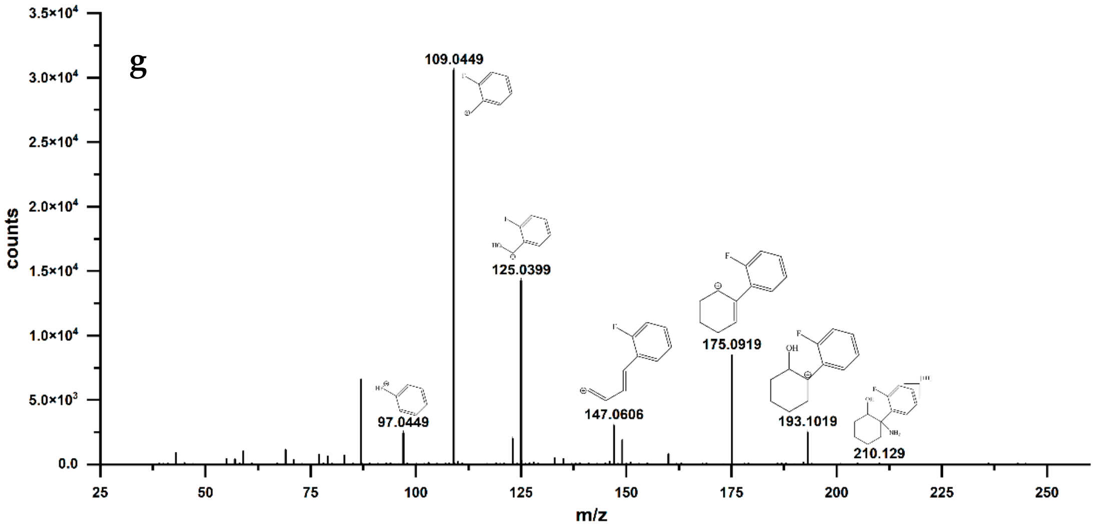
g
counts
m/z
109.0449
125.0399
175.0919
147.0606
193.1019
97.0449
210.129

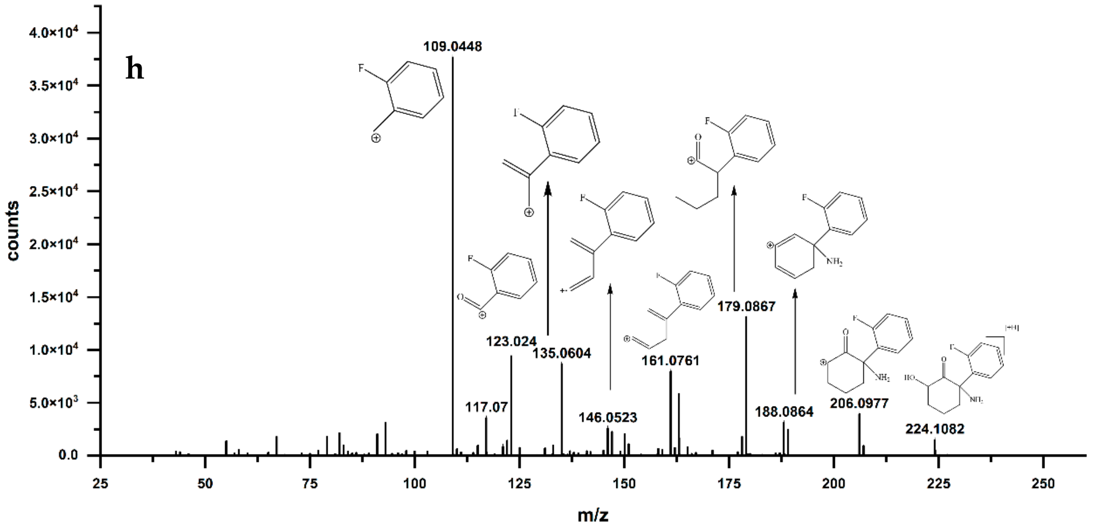
h
counts
m/z
109.0448
123.024
135.0604
179.0867
117.07
146.0523
161.0761
188.0864
206.0977
224.1082

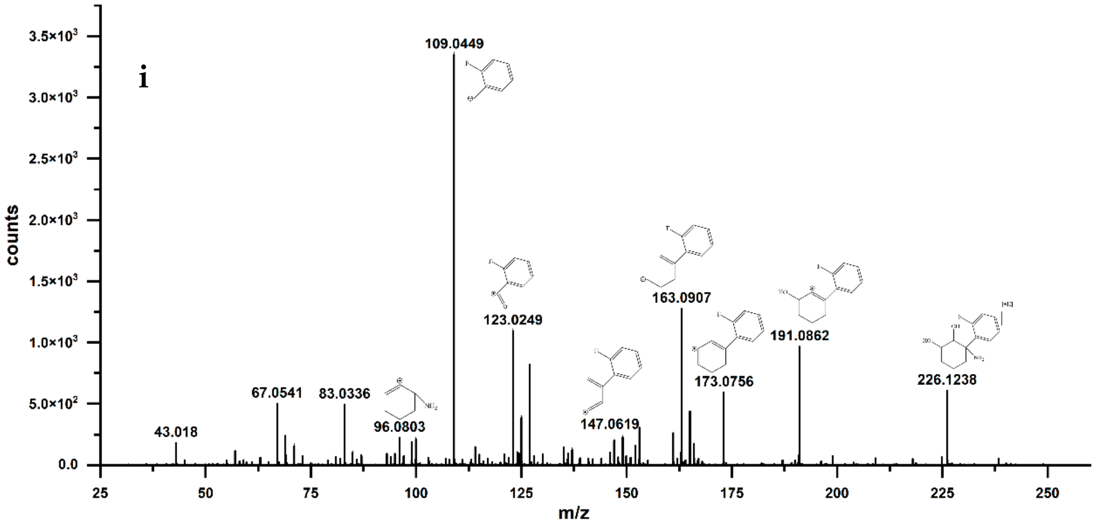
i
counts
m/z
109.0449
123.0249
163.0907
191.0862
173.0756
226.1238
147.0619
67.0541
83.0336
96.0803
43.018

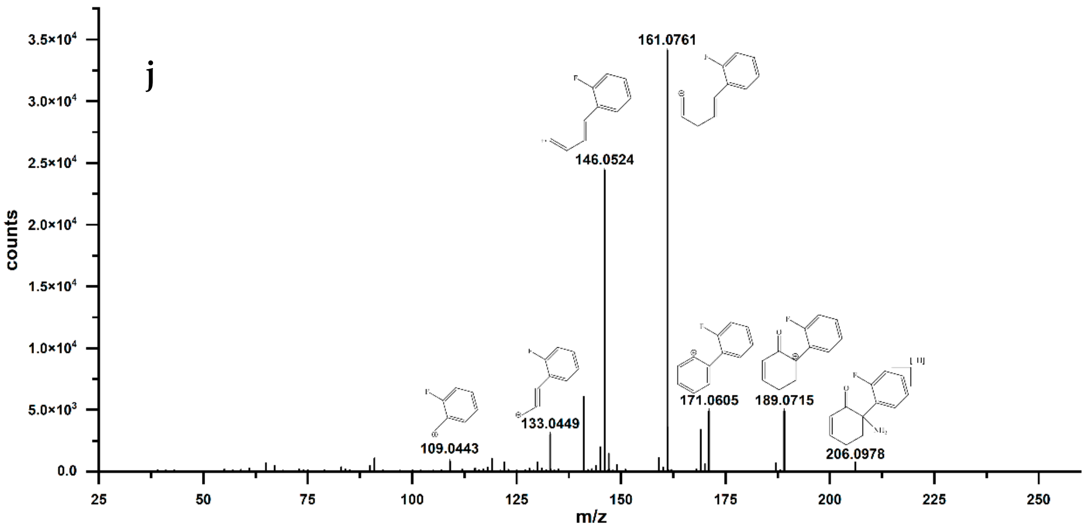

Figure S1. Secondary mass spectra and fragmental information of main metabolites of 2-FDCK.(a) 2-FDCK; (b) M01; (c) M03; (d) M08; (e) M09; (f) M11; (g) M07; (h) M17; (i) M02; (j) M05.

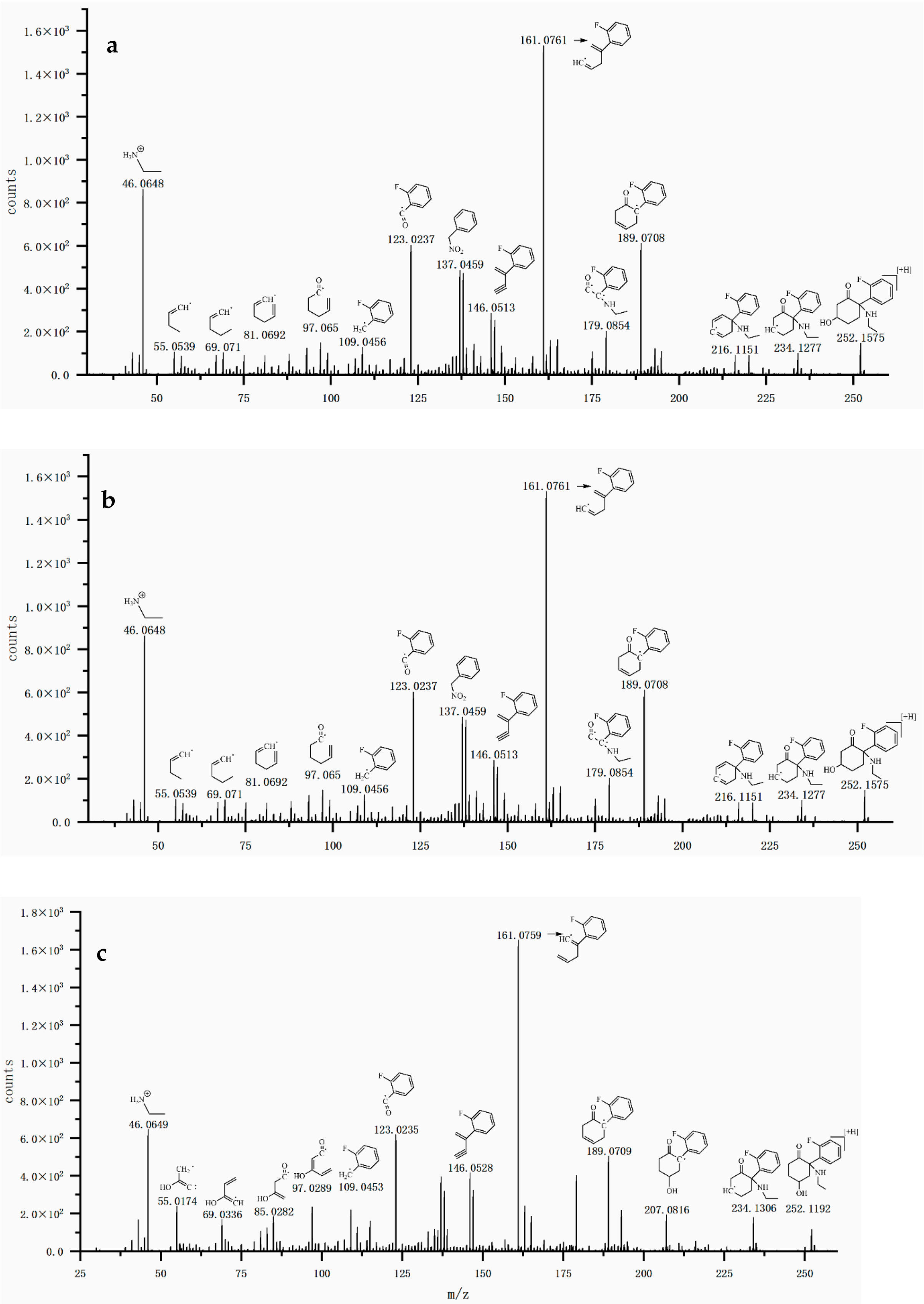
a
counts
161.0761
46.0648
123.0237
137.0459
146.0513
189.0708
179.0854
55.0539
69.071
81.0692
97.065
109.0456
216.1151
234.1277
252.1575
b
counts
161.0761
46.0648
123.0237
137.0459
146.0513
189.0708
179.0854
55.0539
69.071
81.0692
97.065
109.0456
216.1151
234.1277
252.1575
c
counts
161.0759
46.0649
123.0235
146.0528
189.0709
55.0174
69.0336
85.0282
97.0289
109.0453
207.0816
234.1306
252.1192
m/z

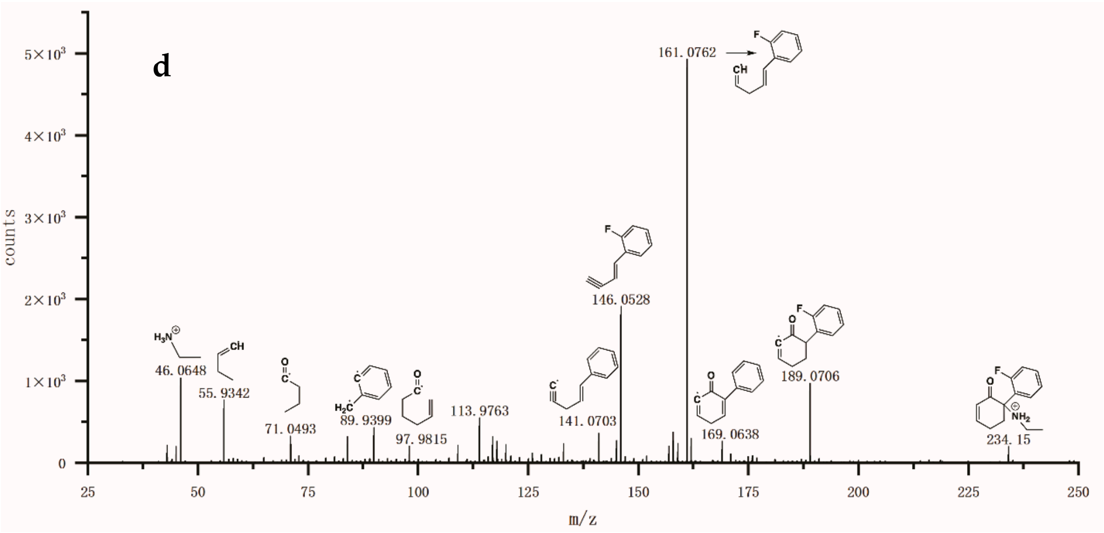
d
counts
m/z
46. 0648
55. 9342
71. 0493
89. 9399
97. 9815
113. 9763
141. 0703
146. 0528
161. 0762
169. 0638
189. 0706
234. 15

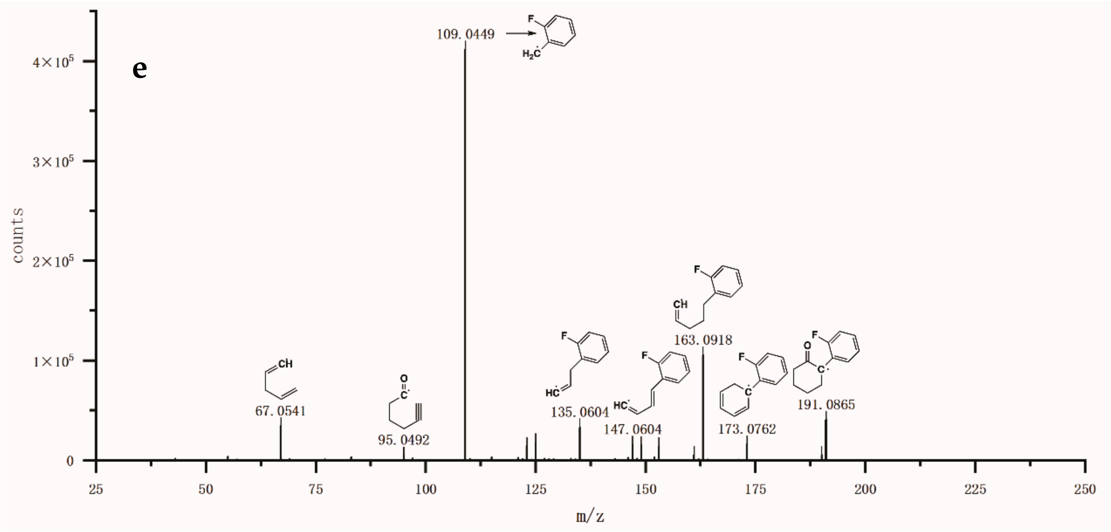
e
counts
m/z
109. 0449
67. 0541
95. 0492
135. 0604
147. 0604
163. 0918
173. 0762
191. 0865

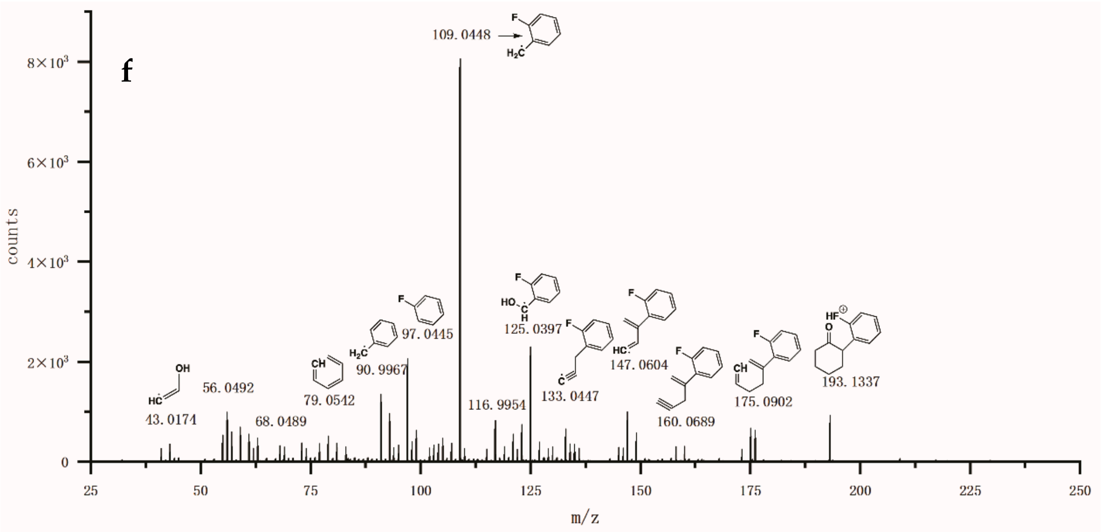
f
counts
m/z
109. 0448
43. 0174
56. 0492
68. 0489
79. 0542
90. 9967
97. 0445
116. 9954
125. 0397
133. 0447
147. 0604
160. 0689
175. 0902
193. 1337

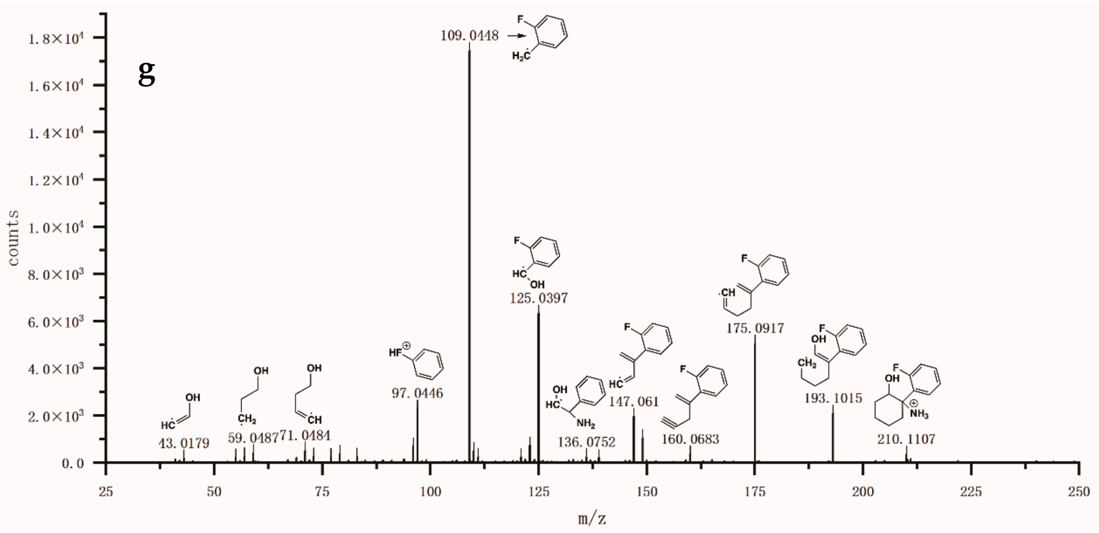
g
counts
m/z
109.0448
125.0397
175.0917
147.061
193.1015
97.0446
43.0179
59.0487
71.0484
136.0752
160.0683
210.1107

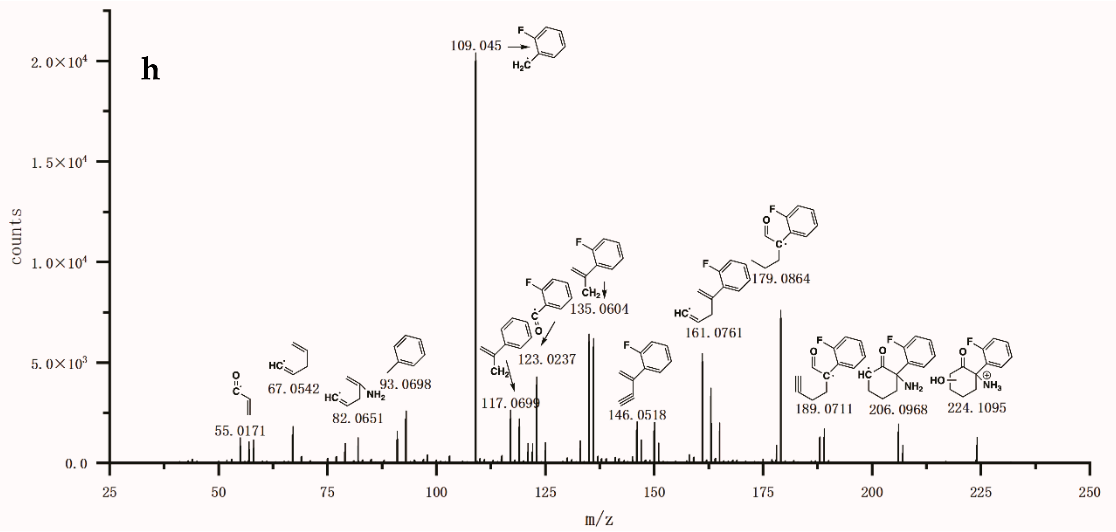
h
counts
m/z
109.045
135.0604
123.0237
117.0699
161.0761
179.0864
146.0518
55.0171
67.0542
82.0651
93.0698
189.0711
206.0968
224.1095

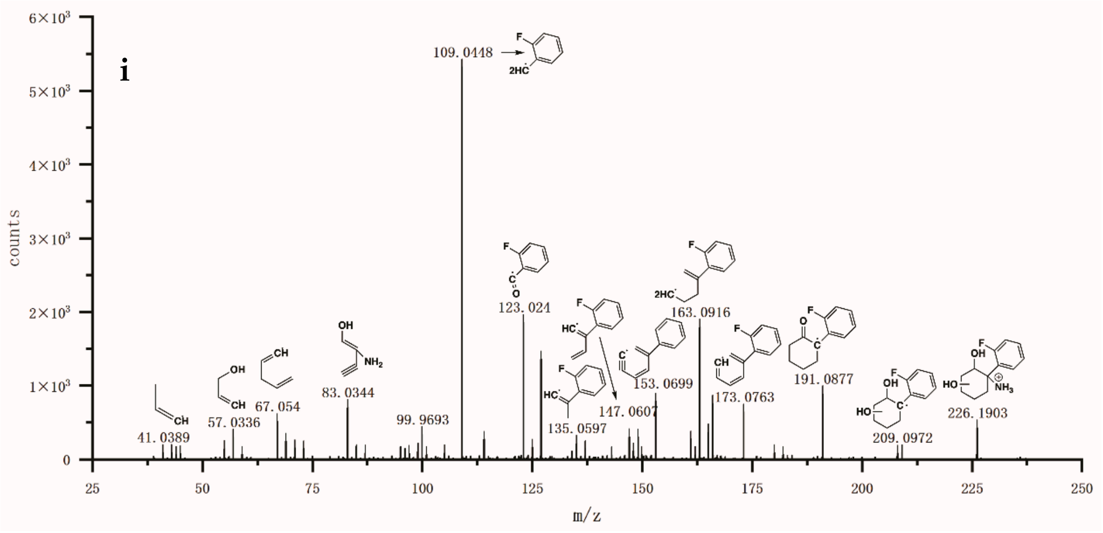
i
counts
m/z
109.0448
123.024
163.0916
153.0699
147.0607
135.0597
173.0763
191.0877
41.0389
57.0336
67.054
83.0344
99.9693
209.0972
226.1903

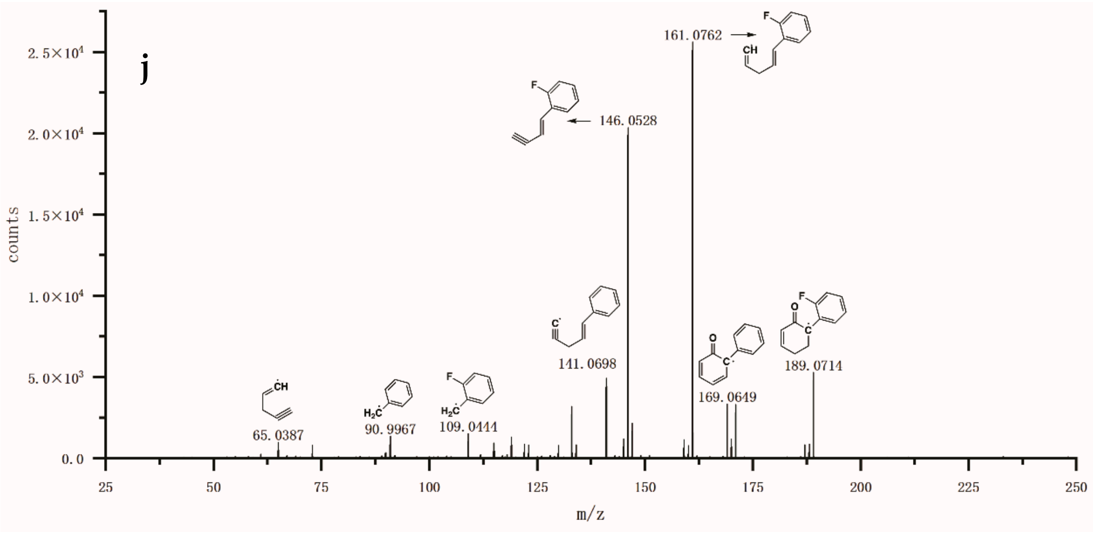

Figure S2. Secondary mass spectra and fragmental information of main metabolites of 2-FXE. (a) 2-FXE (b) M01-N; (c) M03-N; (d) M08-N; (e) M09-N; (f) M11-N; (g) M07-N; (h) M17-N; (i) M02-N; (j) M05-N.

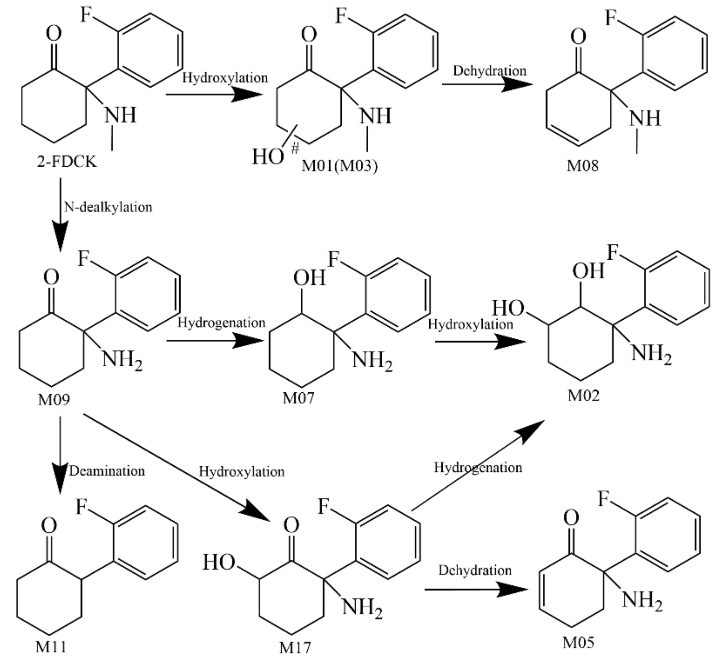

a

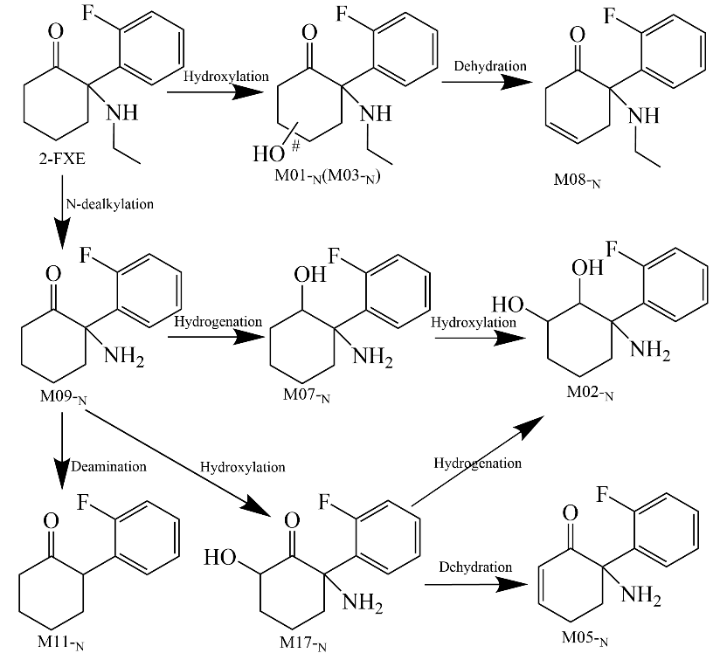

b

Figure S3. Main metabolic pathways of 2-FDCK and 2-FXE. (a) 2-FDCK; (b) 2-FXE.
